# Patients with chronic lymphocytic leukaemia (CLL) differ in the pattern of CTLA-4 expression on CLL cells: the possible implications for immunotherapy with CTLA-4 blocking antibody

**DOI:** 10.1007/s13277-015-4217-1

**Published:** 2015-10-21

**Authors:** Lidia Ciszak, Irena Frydecka, Dariusz Wolowiec, Aleksandra Szteblich, Agata Kosmaczewska

**Affiliations:** 1 Laboratory of Immunopathology, Department of Experimental Therapy, Ludwik Hirszfeld Institute of Immunology and Experimental Therapy, Polish Academy of Sciences, R. Weigla 12, 53-114 Wroclaw, Poland; 2 Department and Clinic of Haematology, Blood Neoplasms, and Bone Marrow Transplantation, Wroclaw Medical University, L. Pasteura 4, 50-367 Wroclaw, Poland

**Keywords:** Chronic lymphocytic leukaemia (CLL), CTLA-4 (CD152), Ki67 protein, Proliferation, Apoptosis

## Abstract

Recently, systemic administration of a human monoclonal antibody directed against cytotoxic T lymphocyte-associated antigen 4 (CTLA-4) expressed on circulating T cells in patients with chronic lymphocytic leukaemia (CLL) has been considered. Also, CLL cells have been shown to express CTLA-4, increased levels of which in the leukaemic compartment are a predictor of good clinical outcome. Since both CLL and Treg microenvironment cells can be targeted by the CTLA-4 blocking antibody in this immunotherapy approach, the investigation of the functional effect of CTLA-4 blockade on CLL cells might be of potential clinical relevance. The main aim of this study was to examine the effect of CTLA-4 blockade on proliferation activity and apoptosis of CLL cells in patients with low and high CTLA-4 expression. We found that in the high CTLA-4-expressing CLL group, CTLA-4 blockade on the CLL cell surface resulted in a significant increase in the median percentages of Ki67^+^ cells and a tendency to decrease in the proportion of apoptotic cells. In contrast, in the low CTLA-4 expressors, CTLA-4 blockade did not affect the proliferation activity or the frequency of apoptosis. This study reports for the first time the different effect of CTLA-4 blockade on CLL cells in CLL patients depending on the levels of CTLA-4 expression. CTLA-4 blockade seems to induce pro-survival signals in leukaemic cells from CLL patients exhibiting high CTLA-4 expression, suggesting that an immunotherapy approach based on the systemic use of monoclonal anti-CTLA-4 antibodies could be an unfavourable strategy for some CLL patients.

## Introduction

Chronic lymphocytic leukaemia (CLL) is the most frequent type of leukaemia recognised in North America, Europe and Australasia, accounting for about one third of all cases of adult leukaemia [1–3]. It is a disease of senior age, since the median age at diagnosis is close to 70 years [2]. The diagnosis of CLL is based on the identification of a monoclonal lymphocytosis of morphologically mature CD19^+^CD5^+^ cells, which spread to the peripheral blood, bone marrow and secondary lymphoid organs such as lymph nodes [2–4]. With progression of the disease, lymphadenopathy, splenomegaly, hepatomegaly, anaemia and thrombocytopenia can occur [2, 3]. Recent studies indicate that the progression and evolution of CLL can be associated with increased proliferation of CLL cells in proliferation centres (PCs) of lymph nodes and bone marrow resulting from the interaction with the microenvironment [5–9].

CLL patients display significant clinical heterogeneity [10, 11]. In some patients, the disease remains stable for many years or develops very slowly [10, 11]. These patients live for prolonged periods without any therapy or may never need treatment in their lifetimes. In others, the disease progresses quickly toward more advanced stages [10, 11]. These patients require early therapy and die relatively rapidly despite aggressive treatment. Due to the variable clinical presentation and evolution of CLL, it is important to identify prognostic factors which will allow identification of CLL patients with low and high risk of disease progression.

A protein recently considered as a prognostic factor in CLL is cytotoxic T lymphocyte-associated antigen 4 (CTLA-4). It has been reported that CTLA-4 acts as a negative regulator of T cell activation and its engagement results in suppression of cell proliferation and cytokine production [12, 13], whereas in Treg cells, CTLA-4 is responsible for the suppressor function of these cells [14]. The increased expression of the CTLA-4 molecule found in the T cell compartment in CLL patients seems to be an unfavourable factor, because it specifically inhibits anti-tumour immunity in these patients [15–17]. Therefore, monoclonal antibodies blocking CTLA-4 on the surface of T cells including Tregs from CLL patients can augment anti-tumour immunity, and CTLA-4 blockade may represent a therapeutic opportunity to enhance the immune responses against autologous leukaemia cells [17]. In fact, previous studies have shown that CTLA-4 blockade enhanced effector T cell function in CLL patients [17]. However, the administration of CTLA-4 blocking antibody in CLL might be complicated by the fact that the CTLA-4 molecule was found to be also expressed on B lymphocytes including leukaemic cells [18–21]. The CTLA-4 molecule is overexpressed in peripheral blood CLL cells compared to normal B lymphocytes from healthy individuals, but the level of its expression is very variable among CLL patients [18, 20]. It has been found that the level of expression of the *CTLA-4* gene in CLL cells is a reliable indicator predicting survival and treatment requirements for CLL patients, since its higher activity in these cells is associated with good clinical outcome, and its lower expression is correlated with a significantly short time to treatment and poor prognosis [19]. In addition, a polymorphism of the *CTLA-4* gene may confer susceptibility to CLL [22]. It was found that the presence of the T allele in the polymorphic site *-*319C>T of the *CTLA-4* gene increased the risk of CLL and, in addition, was correlated with disease progression [22]. Actually, an association between expression of the CTLA-4 molecule in CLL cells and the clinical parameters has been demonstrated [18]. Higher expression of the CTLA-4 molecule in CLL cells is associated with lower Rai stages and lower leukocyte and lymphocyte count [18]. Our and others’ research indicates that CTLA-4 might regulate G1 phase progression [18, 20] and inhibit the proliferation and survival of leukaemic cells [21]. Based on all these findings, systemic administration of a CTLA-4 blocking antibody would affect not only T cell, but also CLL cell biology [18–21].

As we recently reported variability of CTLA-4 expression and its functional relevance in the CLL compartment [19–21], we decided to investigate whether CLL patients differ in the pattern of CLL cell responses to CTLA-4 blockade. The main aim of this study was to investigate the proliferation activity and apoptosis of CLL cells after blockade of the CTLA-4 molecule on the surface of leukaemic cells. A control stimulating culture without CTLA-4 blockade was simultaneously performed. All mentioned experiments were also performed in normal B lymphocytes isolated from peripheral blood of healthy individuals. An assessment of the effect of CTLA-4 blockade on proliferation and apoptosis of CLL cells may contribute to determining whether systemic administration of monoclonal anti-CTLA-4 antibodies is a favourable and safe therapeutic strategy for all CLL patients. As some phase I/II clinical trials using systemic administration of CTLA-4 blockade in haematologic malignancies, including CLL, showed durable clinical responses in a relatively low proportion of patients [23], we hope that the results of our in vitro blocking experiments on CLL cells may provide new insights into the safety and efficacy of this potential therapeutic approach in CLL. To the best of our knowledge, such experiments carried out on CLL cells are lacking so far.

## Materials and methods

### Patients and healthy donors

The study design was approved by the local Bioethical Committee at the Medical University of Wroclaw, Poland, and is in accordance with the Helsinki Declaration of 1975. All participants gave written informed consent after the purpose of the study was explained to them. Thirty-eight previously untreated CLL patients of the Clinic of Haematology, Blood Neoplasms, and Bone Marrow Transplantation, Wroclaw Medical University, Poland, were enrolled in this study. In each of them, the diagnosis was established according to generally accepted criteria including the absolute peripheral blood lymphocytosis ≥5 × 10^9^/L and the co-expression of CD5, CD19 and CD23 antigens on malignant cells. The disease stages were determined according to the Rai classification. Clinical and laboratory features are presented in Table 1.

**Table 1:** Clinical characteristics of CLL patients

Characteristics	Value
Number of patients	38
Gender (female/male)	15/23
Age (years, mean and SD)	69.0 ± 11.2
Rai stage	
0	14
I	7
II	6
III	4
IV	7
WBC count (1 × 10^9^/l)	72.2 ± 98.4
Lymphocyte count (1 × 10^9^/l)	66.5 ± 96.8
Hb level (g/dl)	12.9 ± 1.9
Platelet count (1 × 10^9^/l)	135.2 ± 55.1
LDH (U/l)	206.8 ± 55.3
β2 microglobulin (mg/l)	3.3 ± 1.4

For age and clinical parameters, the mean values and standard deviation (SD) were presented

Leukocyte-enriched fractions of peripheral blood donated by 15 healthy volunteers matched for age and sex with the CLL patients were purchased from the Regional Centre of Blood Donation and Treatment in Wroclaw, Poland.

### Cell isolation and separation procedures

Peripheral blood mononuclear cells (PBMCs) were separated from heparinised freshly drawn peripheral venous blood of CLL patients and healthy controls by buoyant density gradient centrifugation on Lymphoflot (Bio-Rad Medical Diagnostics GmbH, Dreieich, Germany) and washed three times in phosphate-buffered saline (PBS) (without Ca^2+^ and Mg^2+^). The PBMCs were suspended in 95 % foetal calf serum (CytoGen GmbH, Sinn, Germany) containing 5 % DMSO (Sigma-Aldrich, St. Gallen, Switzerland) and stored in liquid nitrogen until used.

CLL cells were isolated from PBMCs by negative selection using EasySep Human B Cell Enrichment Kit without CD43 Depletion (STEMCELL Technologies Inc, Vancouver, Canada) according to the manufacturer’s instructions. Following this separation procedure, more than 98 % of the resulting cell population was CD19^+^CD5^+^ as assessed by flow cytometry using anti-CD19 and anti-CD5 monoclonal antibodies (mAbs) (Becton Dickinson, BD Biosciences, San Diego, USA). Normal B cells from healthy individuals were isolated from PBMCs by negative selection using the EasySep Human B Cell Enrichment Kit (STEMCELL Technologies Inc, Vancouver, Canada) according to the manufacturer’s instructions, achieving above 98 % purity as assessed by flow cytometry using anti-CD19 mAbs.

### Culture conditions

Purified normal CD19^+^ lymphocytes or CLL cells were suspended at 1 × 10^6^ cells/ml in RPMI-1640 medium (Gibco, Paisley, UK), supplemented with 10 % foetal calf serum (CytoGen GmbH, Sinn, Germany), 2 mmol/l l-glutamine and 50 μg/ml gentamycin (KRKA-Poland, Warsaw, Poland), and cultured using 24-well U-bottom culture plates (Nunc GmbH & Co. KG, Langenselbold, Germany) at 37 °C in a 5 % CO_2_ humidified atmosphere for 24 and 72 h either in medium alone or together with 1 μM DSP30 (5′-TCGTCGCTGTCTCCGCTTCTTCTTGCC-3′) (TIB MOLBIOL, Berlin, Germany) [24] and 100 U/ml rIL-2 (Eurocetus, Amsterdam, The Netherlands) [25]. For the blocking experiment, purified CLL cells and normal CD19^+^ lymphocytes were cultured with 1 μM DSP30 and 100 U/ml IL-2 with the blocking anti-CTLA-4 mAbs (50 μg/ml) (BD Pharmingen, BD Biosciences, San Diego, USA) [26] or control IgG2 (50 μg/ml) (BD Pharmingen, BD Biosciences, San Diego, USA).

### Immunostaining of CTLA-4, Ki67 protein and flow cytometric analysis

The expression of these molecules was studied in purified CLL cells and normal CD19^+^ lymphocytes before and after 24- and 72-h culture by a single immunostaining method.

Briefly, for detection of surface expression of the CTLA-4 molecule, the cells were washed twice in PBS (without Ca^2+^ and Mg^2+^), divided into tubes at a concentration of 5 × 10^5^ cells per tube and incubated with anti-CTLA-4 (CD152)/retinal pigment epithelium (RPE) mAbs (BD Pharmingen, BD Biosciences, San Diego, USA) for 30 min at 4 °C in the dark. Excess unbound antibodies were removed by two washes with PBS. Following these washes, the cells were resuspended in PBS and analysed by flow cytometry using a FACSCalibur flow cytometer (Becton Dickinson, BD Biosciences, San Diego, USA). For determination of intracellular CTLA-4 expression, the cells were first fixed for 10 min at room temperature in 2 % paraformaldehyde (Fluka, Sigma-Aldrich, Buchs, Germany), washed in PBS and incubated for 10 min at room temperature in BD Permeabilizing Solution 2 (Becton Dickinson, BD Biosciences, San Diego, USA) according to the manufacturer’s instructions. Then, the cells were incubated with anti-CTLA-4 (CD152)/R-phycoerythrin (R-PE) mAbs for 30 min at 37 °C in the dark.

Ki67 protein was detected by staining of the cells with anti-Ki67/fluorescein isothiocyanate (FITC) mAbs (BD Pharmingen, BD Biosciences, San Diego, USA) after fixation and permeabilisation as described in the case of intracellular detection of the CTLA-4 molecule.

Negative controls were always done by omitting the mAbs and by incubating the cells with mouse Ig of the same isotype as the mAbs conjugated with RPE or FITC. At least 10,000 events per sample were analysed. The results were expressed as the proportion of CTLA-4- or Ki67-positive cells. The CellQuest program was used for statistical analysis of the acquired data.

### Apoptosis

Before and after cell culture, the apoptosis was determined by flow cytometry using the In Situ Cell Death Detection Kit, Fluorescein (Roche Diagnostics GmbH, Mannheim, Germany) based on a terminal deoxynucleotidyl transferase (TdT)-mediated dUTP nick-end labelling (TUNEL) assay. At least 10,000 events per sample were analysed. The CellQuest program was used for statistical analysis of the acquired data.

### Statistical analysis

Statistical analyses of the clinical data and laboratory findings were conducted using Statistica 10.0 or PQStat software. For clinical parameters, the mean values and standard deviation were calculated. For all other analysed variables, the median values and 25th and 75th interquartile range were calculated.

All collected data were examined for normal distribution and for homogeneity of variances using the Shapiro-Wilk test and Levene’s test, respectively. If data were normally distributed and had homogeneous variances, the comparisons between both studied groups of CLL patients and healthy individuals were performed using the one-factor analysis of variance (ANOVA) followed by a post hoc test (Scheffe *F* test). To test the effects of culture and CTLA-4 blockade on analysed variables, the repeated measures ANOVA and the Student’s *t* test for dependent samples were used. If data were not normally distributed and/or had heterogeneous variances, the non-parametric Kruskal-Wallis one-way ANOVA by rank, the Friedman ANOVA test followed by a post hoc test (Dunn test) and the non-parametric Wilcoxon signed-rank test were applied. In all analyses, differences were considered significant when *P* ≤ 0.05.

## Results

### Surface and intracellular expression of the CTLA-4 molecule in freshly drawn CLL cells and normal CD19^+^ lymphocytes

Since CTLA-4 is transiently expressed on the cell surface and is predominantly located in intracellular compartments due to constitutive internalisation from the plasma membrane [27], we determined both surface (sCTLA-4) and cytoplasmic (cCTLA-4) expression of this molecule. We found significantly elevated expression of CTLA-4 in patients’ CLL cells compared to B cells from healthy controls (*p* ≤ 0.001; data not shown). Also worthy of note was variability of both surface and cytoplasmic CTLA-4 expression in the CLL group, ranging from 3.5 to 57.1 % and from 10.1 to 74.1 %, respectively. Based on the fact that the surface CTLA-4 is of functional significance as a regulator of cell activation, we divided the patients into low and high expressors of the CTLA-4 molecule, taking as a cut-off value the median percentage of sCTLA-4-positive CLL cells (32.8 %). Accordingly, in the high CTLA-4 expressors, the median proportions of both sCTLA-4-positive cells and cCTLA-4-positive cells were markedly higher compared to the low CTLA-4 expressors and healthy individuals (Table 2). In contrast, no differences in the median frequencies of these cells between the low CTLA-4 expressors and healthy volunteers were found. Although the surface as well as cytoplasmic expression of the CTLA-4 molecule in single cells defined as mean fluorescence intensity (MFI) was the highest in the high CTLA-4 expressors and the lowest in the low CTLA-4 expressors, the differences between all studied groups were not statistically significant (data not shown).

**Table 2:** Median proportions and 25th–75th interquartile range of leukaemic cells and normal B cells co-expressing CTLA-4 molecule

Groups	Unstimulated	24-h culture		72-h culture	
		Medium alone	DSP30+rIL-2	Medium alone	DSP30+rIL-2
% sCTLA-4-positive cells					
Low CTLA-4 expressors (*n* = 19)	22.9 [19.2–26.9]	24.5 [16.3–33.8]	22.9 [18.4–28.5]	16.9 [12.5–25.0]	14.9 [10.7–28.0]
High CTLA-4 expressors (*n* = 19)	44.6 [35.4–53.3]	32.0 [27.6–40.7]	26.7 [25.3–29.4]	18.7 [16.6–25.0]	18.9 [17.0–23.2]
Controls (*n* = 15)	18.8 [17.9–20.1]	17.9 [11.4–24.9]	20.9 [13.2–28.5]	11.8 [6.0–14.9]	14.8 [11.1–24.5]
Low vs high CTLA-4 expressors	*P* = 0.0000001	*P* = 0.03	NS	NS	NS
Low CTLA-4 expressors vs controls	NS	NS	NS	NS	NS
High CTLA-4 expressors vs controls	*P* = 0.0000001	*P* = 0.009	NS	NS	NS
% cCTLA-4-positive cells					
Low CTLA-4 expressors (*n* = 19)	29.7 [19.6–39.7]	45.0 [30.1–58.2]	45.3 [36.7–55.1]	29.0 [13.4–43.2]	36.4 [23.6–63.4]
High CTLA-4 expressors (*n* = 19)	54.4 [43.8–65.5]	53.2 [49.6–60.0]	56.9 [51.4–67.6]	46.2 [37.5–53.2]	50.1 [44.1–57.0]
Controls (*n* = 15)	25.7 [8.7–33.1]	30.4 [23.0–37.3]	26.2 [21.3–40.7]	14.1 [9.6–21.3]	19.1 [4.5–27.0]
Low vs high CTLA-4 expressors	*P* = 0.005	NS	NS	NS	NS
Low CTLA-4 expressors vs controls	NS	NS	NS	NS	*P* = 0.004
High CTLA-4 expressors vs controls	*P* = 0.00004	*P* = 0.002	*P* = 0.0004	*P* = 0.0007	*P* = 0.000005

### Proliferation activity and apoptosis of freshly drawn leukaemic cells from CLL patients with low and high CTLA-4 expression and normal B lymphocytes

Since CTLA-4 is known as an anti-proliferative factor [12, 13, 28], we examined whether freshly drawn CLL cells from the two studied groups of CLL patients differ regarding the proliferation activity; therefore, the expression of Ki67 protein in CLL cells was estimated. In both studied groups of CLL patients, the proliferation activity was low and comparable to that found in healthy individuals (Table 3).

**Table 3:** Median proportions and 25th–75th interquartile range of leukaemic cells and normal B cells co-expressing Ki67

Groups	Unstimulated	24-h culture		72-h culture	
		Medium alone	DSP30+rIL-2	Medium alone	DSP30+rIL-2
% Ki67-positive cells					
Low CTLA-4 expressors (*n* = 19)	2.1 [0.3–4.5]	2.2 [1.3–4.3]	10.6 [3.8–28.8]	1.5 [1.1–1.9]	9.2 [4.0–21.6]
High CTLA-4 expressors (*n* = 19)	3.3 [1.6–3.6]	2.3 [1.3–3.6]	10.2 [2.5–15.1]	2.2 [1.6–3.0]	9.3 [5.9–12.0]
Controls (*n* = 15)	1.0 [0.0–2.8]	0.0 [0.0–0.2]	13.2 [10.2–15.0]	0.4 [0.0–3.3]	13.4 [11.3–17.5]
Low vs high CTLA-4 expressors	NS	NS	NS	NS	NS
Low CTLA-4 expressors vs controls	NS	NS	NS	NS	NS
High CTLA-4 expressors vs controls	NS	NS	NS	NS	*P* = 0.05

As CTLA-4 is involved in the regulation of cell survival [21, 29], we also investigated whether freshly drawn CLL cells from the two studied groups of CLL patients differ in terms of the apoptosis rate. We did not detect any apoptotic cells either in studied group of CLL patients or in healthy individuals (Table 4).

**Table 4:** Median proportions and 25th–75th interquartile range of apoptotic leukaemic cells and normal B cells

Groups	Before cell culture	72-h culture	
		Medium alone	DSP30+rIL-2
% Apoptotic cells			
Low CTLA-4 expressors (*n* = 19)	0.0 [0.0–0.0]	34.3 [24.9–40.5]	44.4 [33.6–58.4]
High CTLA-4 expressors (*n* = 19)	0.0 [0.0–0.0]	19.2 [13.8–24.6]	51.4 [29.6–64.0]
Controls (*n* = 15)	0.0 [0.0–0.0]	26.2 [22.4–28.7]	49.3 [39.9–67.1]
Low vs high CTLA-4 expressors	NS	*P* = 0.02	NS
Low CTLA-4 expressors vs controls	NS	NS	NS
High CTLA-4 expressors vs controls	NS	NS	NS

### Effect of ex vivo stimulation on expression of the CTLA-4 molecule in leukaemic cells from CLL patients with low and high CTLA-4 expression, as well as normal CD19^+^ cells

**Fig. 1:**
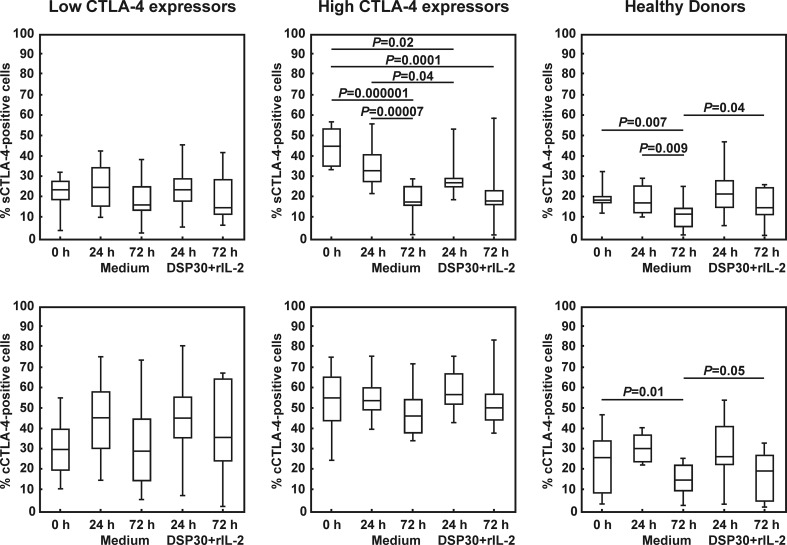
Surface (s) and intracellular expression (c) of CTLA-4 in studied groups of CLL patients and healthy donors before and after cell culture. *Boxes* and *whiskers*: 25th–75th interquartile range and min.-max., respectively; the median is the *central line* in each *box*

**Fig. 2:**
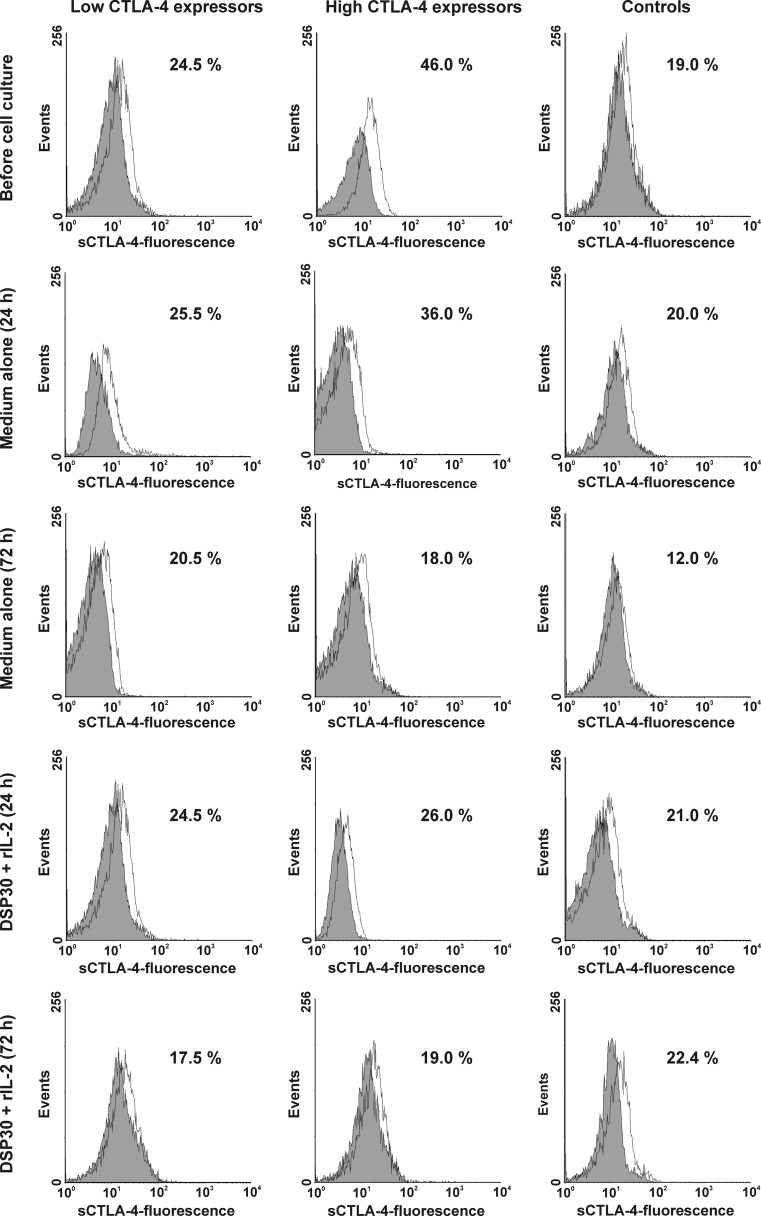
Representative examples of flow cytometric analyses of the surface expression of CTLA-4 (sCTLA-4) in studied groups of CLL patients and healthy controls. Grey histograms represent isotype controls. *Numbers* on histograms represent the percentage of the cells expressing CTLA-4 on the cell surface

Next, we investigated whether leukaemic cells from both studied groups of CLL patients, in particular from the low CTLA-4 expressors, are able to change CTLA-4 expression in response to ex vivo stimulation with DSP30 and rIL-2. As a control, cell culture in medium alone was performed. In the low CTLA-4 expressors, no significant impact of cell culture in medium alone or ex vivo stimulation on the surface expression of the CTLA-4 molecule was found, and the median proportions of sCTLA-4^+^ CLL cells were comparable to those observed in healthy individuals at each time point tested (Table 2, Figs. 1 and 2). In contrast, in the high CTLA-4 expressors, both control and stimulating culture led to a gradual decrease in the median percentages of sCTLA-4-positive leukaemic cells, with the minimum value after 72 h (Table 2, Figs. 1 and 2). In consequence, after 72 h of culture in medium alone as well as after 24 and 72 h of stimulating culture, the median frequencies of sCTLA-4-positive cells in the high CTLA-4 expressors became comparable to those observed in the low CTLA-4 expressors and healthy individuals (Table 2). In healthy volunteers, cell culture in medium alone led to a gradual decrease in the median proportion of CD19^+^sCTLA-4^+^ cells, with the minimum value after 72 h (Figs. 1 and 2). Of note, after 72 h of ex vivo stimulation, the median frequency of sCTLA-4-positive CD19^+^ lymphocytes was markedly higher than in the cell culture in medium alone (Figs. 1 and 2).

**Fig. 3:**
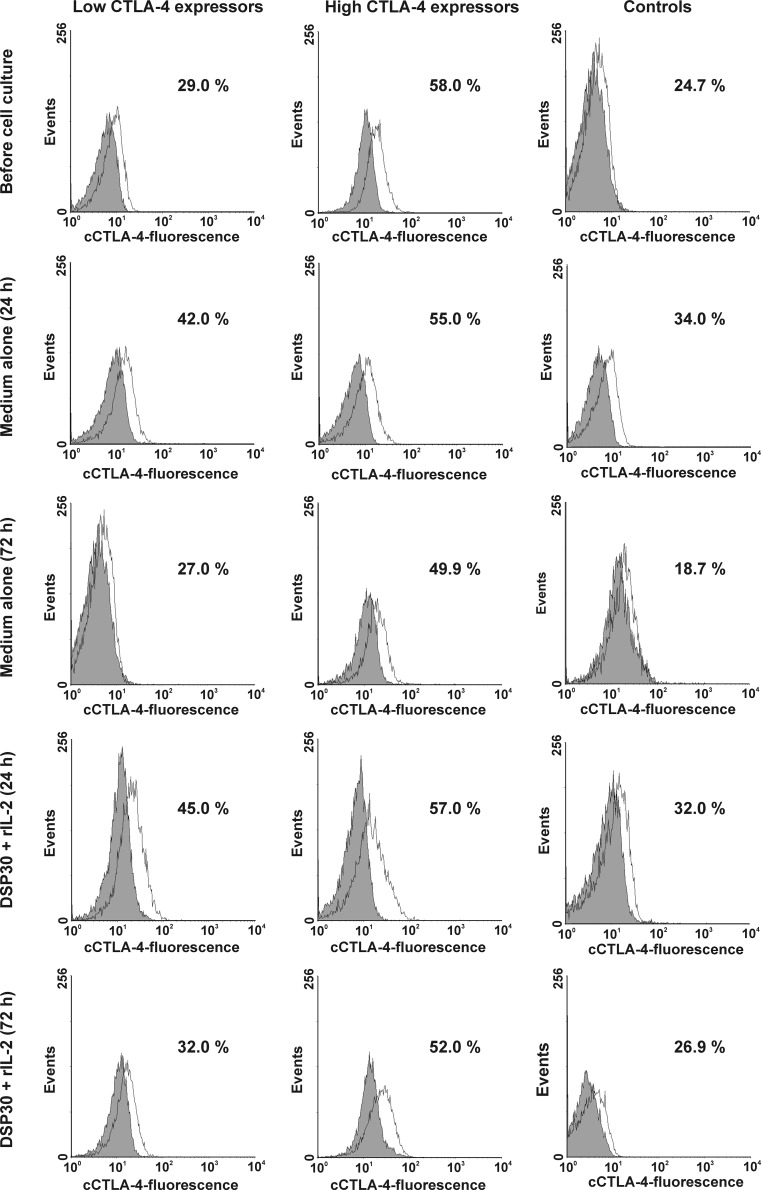
Representative examples of flow cytometric analyses of intracellular expression of CTLA-4 (cCTLA-4) in studied groups of CLL patients and healthy controls. Grey histograms represent isotype controls. *Numbers* on histograms represent the percentage of the cCTLA-4-positive cells

As regard cytoplasmic expression of the CTLA-4 molecule, in both studied groups of CLL patients, no significant impact of control or stimulating culture on the expression of CTLA-4 was observed (Table 2, Figs. 1 and 3). Moreover, no significant differences between the studied groups of CLL patients after cell culture were found (Table 2). It is noteworthy that in the high CTLA-4 expressors, the median percentages of cCTLA-4-positive leukaemic cells remained markedly higher than in healthy volunteers at each time point tested (Table 2), whereas in the low CTLA-4 expressors, the median proportion of cCTLA-4^+^ leukaemic cells became markedly higher than in healthy individuals only after 72 h of ex vivo stimulation (Table 2).

In healthy controls, after 72 h of cell culture in medium alone, we observed a marked decrease in the median percentage of CD19^+^cCTLA-4^+^ cells (Table 2, Figs. 1 and 3). Moreover, after 72 h of ex vivo stimulation, the median proportion of cCTLA-4-positive CD19^+^ lymphocytes was significantly higher than in the cell culture in medium alone (Figs. 1 and 3).

### Effect of ex vivo stimulation on the proliferation capacity of leukaemic cells from CLL patients with low and high CTLA-4 expression and normal B lymphocytes

**Fig. 4:**
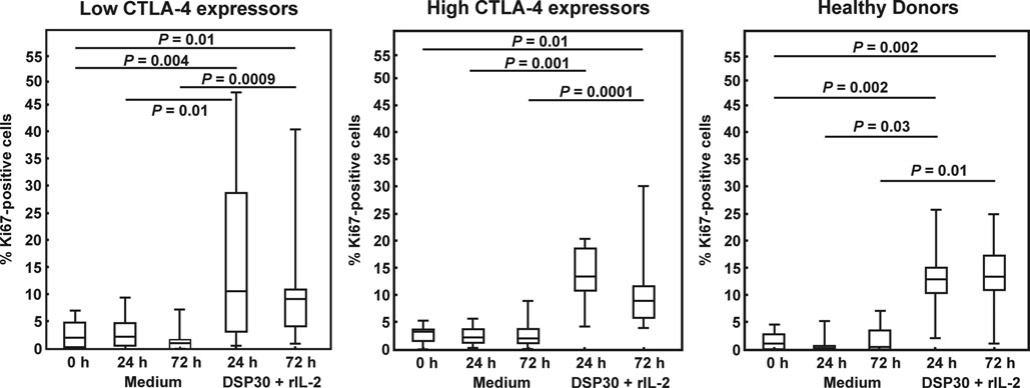
Ki67 expression in studied groups of CLL patients and healthy donors before and after cell culture. *Boxes* and *whiskers*: 25th–75th interquartile range and min.-max., respectively; the median is the *central line* in each *box*

Next, we asked whether the two studied groups of CLL patients differ in terms of proliferation activity following ex vivo stimulation. To answer this question, the expression of Ki67 protein in leukaemic cells after the cell culture in medium alone and in the presence of DSP30+ IL-2 was examined. In both studied groups of CLL patients as well as in healthy individuals, no significant impact of the culture in medium alone on the median proportions of Ki67-positive cells was found (Fig. 4); moreover, the proliferation activity was comparable in both groups of CLL patients and healthy volunteers (Table 3). In contrast, ex vivo stimulation led to a marked increase in the median frequencies of Ki67^+^ cells in all studied groups (Fig. 4). Although the median percentages of Ki67-positive cells did not significantly differ between the studied groups of CLL patients at each time point tested (Table 3), and in the low CTLA-4 expressors the median proportions of Ki67^*+*^ leukaemic cells following ex vivo stimulation were comparable to the corresponding cells in healthy individuals, we observed that in the high CTLA-4 expressors, the median frequency of Ki67-positive leukaemic cells after 72 h of stimulating culture was markedly lower than in healthy controls (Table 3).

### Effect of ex vivo stimulation on the rate of apoptosis of leukaemic cells from CLL patients with low and high CTLA-4 expression and normal B lymphocytes

**Fig. 5:**
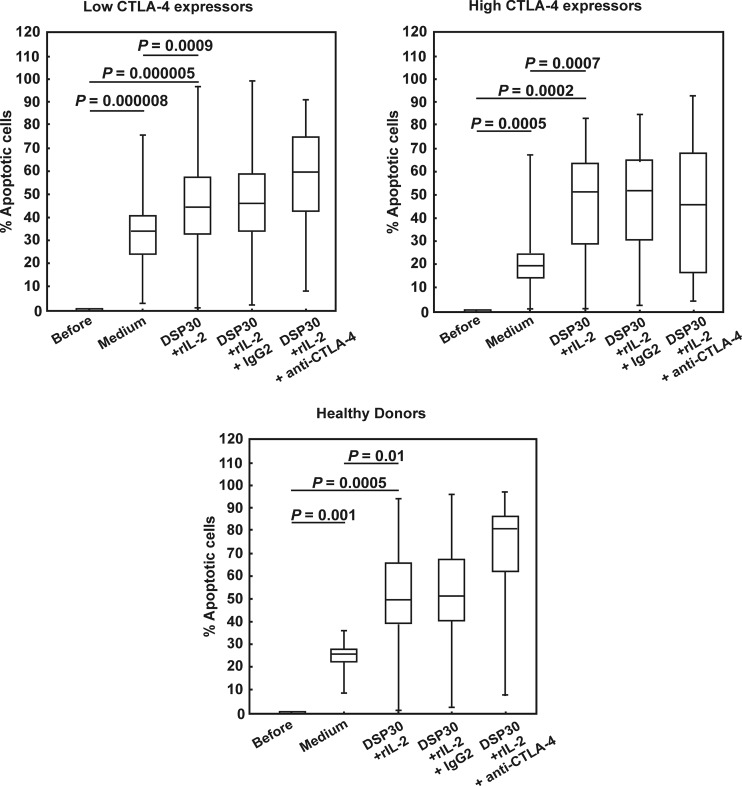
Apoptosis in studied groups of CLL patients and healthy donors before and after cell culture. *Boxes* and *whiskers*: 25th–75th interquartile range and min.-max., respectively; the median is the *central line* in each *box*

**Fig. 6:**
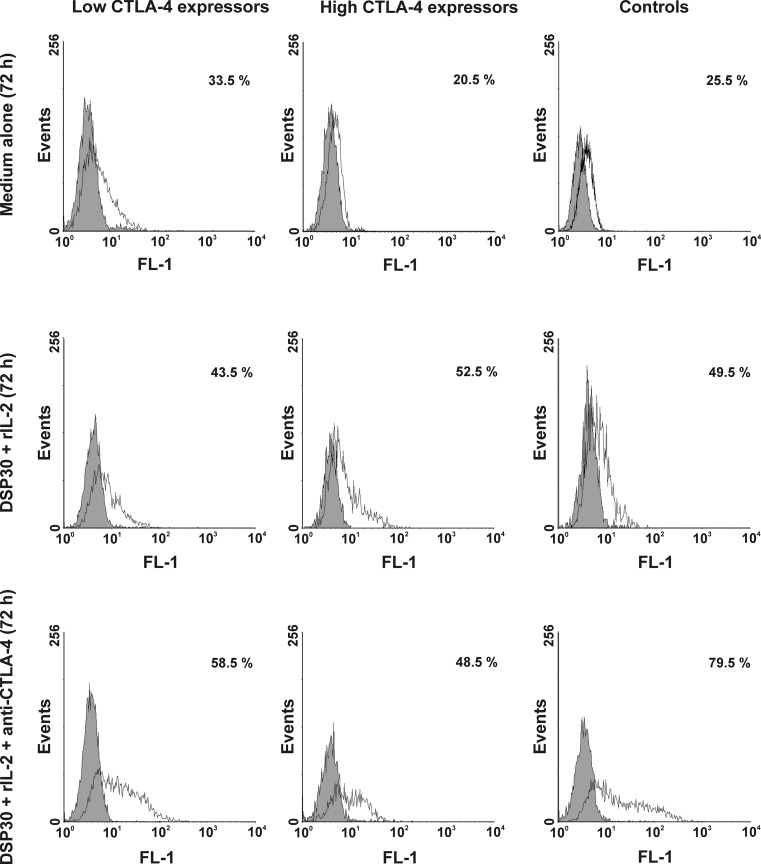
Representative examples of flow cytometric analyses of apoptosis in studied groups of CLL patients and healthy controls. Grey histograms represent isotype controls. *Numbers* on histograms represent the percentage of the apoptotic cells

Simultaneously with proliferation activity, we assessed the apoptosis rate of the studied cells under non-stimulating and stimulating conditions. In both groups of CLL patients as well as in healthy volunteers, we observed a marked increase in the median frequencies of apoptotic cells after culture in medium alone (Table 4, Fig. 5). Of note, the highest increase in the apoptotic cell frequencies was seen in the low CTLA-4 expressors, whereas the smallest increase was found in the high CTLA-4 expressors (Table 4, Fig. 5). In consequence, the median proportion of apoptotic cells after 72 h of culture in medium alone in the low CTLA-4 expressors was significantly higher compared with the high CTLA-4 expressors (Table 4). Ex vivo stimulation led to a further increase in the median frequencies of apoptotic cells to comparable levels in all studied groups (Table 4, Figs. 5 and 6).

### Effect of CTLA-4 blockade on proliferation activity of leukaemic cells from CLL patients with low and high CTLA-4 expression and normal B lymphocytes

**Fig. 7:**
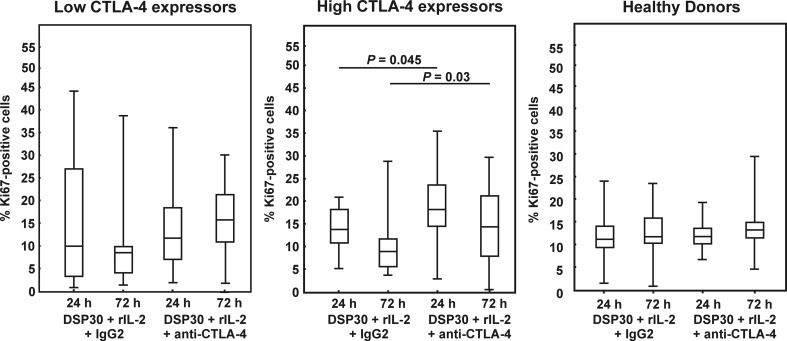
Ki67 expression in studied groups of CLL patients and healthy donors after blocking culture. *Boxes* and *whiskers*: 25th–75th interquartile range and min.-max., respectively; the median is the *central line* in each *box*

**Fig. 8:**
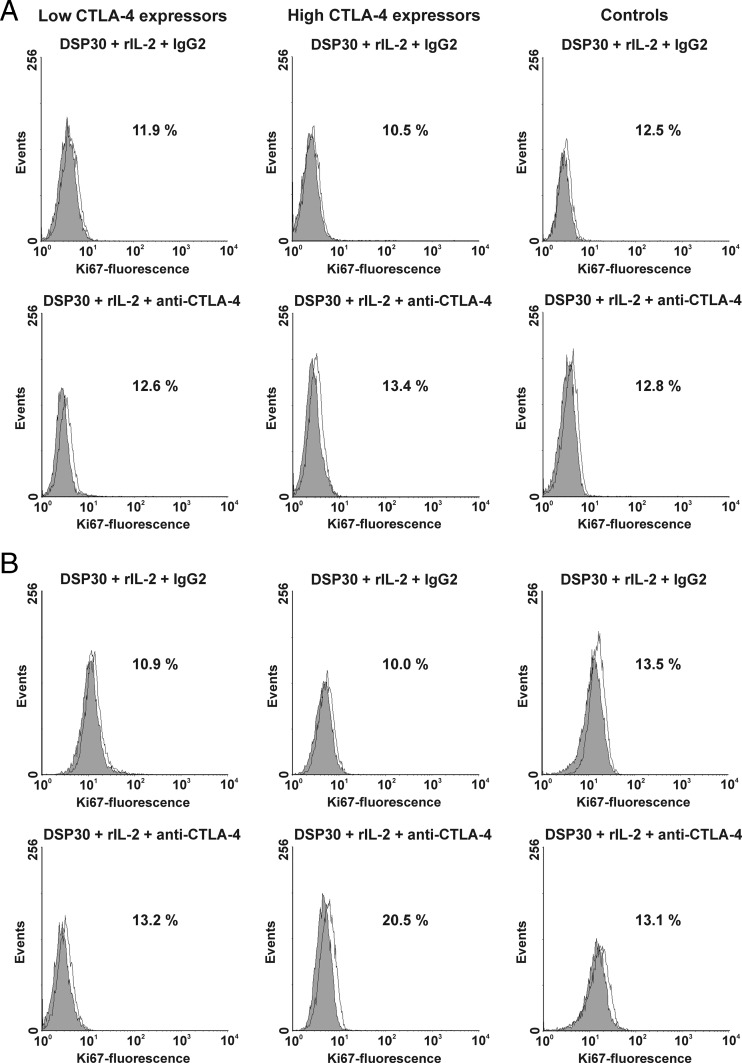
Effect of CTLA-4 blockade on Ki67 expression in studied groups of CLL patients and healthy controls. **a** Histograms show representative data of flow cytometric analyses of the expression of Ki67 protein after 24 h of blocking culture. **b** Histograms show representative data illustrating expression of Ki67 protein after 72 h of blocking culture. Grey histograms represent isotype controls. *Numbers* on histograms represent the percentage of cells expressing Ki67

To find out whether CTLA-4 blockade on the cell surface would affect the proliferation activity, we estimated the median proportions of Ki67-positive cells after cell culture in the presence of DSP30, rIL-2 and the blocking anti-CTLA-4 mAb in all studied groups. In the low CTLA-4 expressors as well as in healthy individuals, no significant impact of CTLA-4 blockade on the median percentages of Ki67^+^ cells was observed (Figs. 7 and 8). In contrast, in the high CTLA-4 expressors, CTLA-4 blockade led to a marked increase in proliferation activity after 24 and 72 h of culture (Figs. 7 and 8), and the median proportion of Ki67-positive cells after 72 h of blocking culture in this group of CLL patients was significantly higher than in healthy individuals (*P* = 0.03).

### Effect of CTLA-4 blockade on apoptosis of leukaemic cells from CLL patients with low and high CTLA-4 expression and normal B lymphocytes

Simultaneously with proliferation activity, we decided to explain whether blocking CTLA-4 on the CLL cell surface would affect apoptosis. In the blocking culture, we observed a further increase in the median frequencies of apoptotic cells in the low CTLA-4 expressors and, more importantly, in healthy individuals, although this increase was not statistically significant (Figs. 5 and 6). In contrast, in the high CTLA-4 expressors, a tendency to decrease in the median proportion of apoptotic cells after CTLA-4 blockade was observed (Figs. 5 and 6). In consequence, in the high CTLA-4 expressors, the median percentage of apoptotic cells after CTLA-4 blockade was markedly lower than in healthy volunteers (*P* = 0.03), whereas in the low CTLA-4 expressors, the median proportion of apoptotic cells was comparable to the value found in healthy individuals.

## Discussion

Herein, we report that CLL leukemic cells exhibit high variability in the response to in vitro stimulation and CTLA-4 blockade depending on the pattern of surface CTLA-4 expression. Considering the fact that both CLL cells and T cells, including the Treg microenvironment, can be targeted by CTLA-4 blocking antibody in a possible immunotherapy approach, our current findings may be of potential clinical relevance.

To the best of our knowledge, among the available reports concerning expression of the CTLA-4 molecule in CLL patients [16–21, 30, 31], only four of them concerned CTLA-4 expression and its function in peripheral blood leukaemic B cells [18–21]. Of note, literature data regarding CTLA-4 expression in CLL cells following ex vivo stimulation with CpG oligodeoxynucleotides (ODNs) are lacking so far. The interesting finding of the present study was that only in the high CTLA-4 expressors were the median percentages of freshly isolated leukaemic cells co-expressing CTLA-4 on the surface as well as in intracellular compartments significantly higher than in healthy individuals. In the low CTLA-4 expressors, the median proportions of both sCTLA-4-positive cells and cCTLA-4-positive cells were comparable to those found in healthy individuals, and a tendency to lower expression of the CTLA-4 molecule in single cells was observed. This finding raises the question about the mechanisms underlying the varied levels of CTLA-4 expression in malignant B cells in CLL patients. Based on our previous notion that *CTLA-4* gene polymorphisms may influence CTLA-4 expression level in CLL cells [20, 22], it is possible that the high level of CTLA-4 expression in some of the CLL patients might result from the presence of specific alleles predisposing to upregulation of expression of the CTLA-4 molecule. The other possible explanation is a potential negative feedback loop between CTLA-4 and NFAT1 (nuclear factor of activated T cells; also known as NFATc2) [21]. NFAT1 binds to the promoter of the *CTLA-4* gene and controls its expression [32–34]. The most recent study showed significantly lower expression of NFAT1 in the high CTLA-4 expressors compared to the low CTLA-4 expressors [21]. Moreover, we cannot exclude that in some CLL patients, signals coming from tissue compartments, including the bone marrow and secondary lymphoid organs, suppress the expression of the CTLA-4 molecule. Recently, Mittal et al. [21] showed that in peripheral blood CLL cells co-cultured on endothelial-derived stromal cells and bone marrow-derived stromal cells, expression of the *CTLA-4* gene was down-regulated.

An original finding of this study was that CLL patients also differ regarding the pattern of CTLA-4 expression following cell culture. In the low CTLA-4 expressors, the surface as well as cytoplasmic expression of CTLA-4 remained unchanged under both non-stimulating (cell culture in medium alone) and stimulating conditions (DSP30+rIL-2). In contrast, in the high CTLA-4 expressors, a gradual decrease of surface CTLA-4 expression on leukaemic cells during culture in medium alone was observed. Moreover, in the same group, ex vivo stimulation led to a further significant decrease in the surface expression of the CTLA-4 molecule. Simultaneously, the median proportions of cytoplasmic CTLA-4-positive cells did not change during the cell culture in medium alone or in the presence of DSP30 and rIL-2. These observations seem to suggest that the down-regulation of surface expression of CTLA-4 observed in the high CTLA-4-expressing CLL patients may result from disturbed recycling of the CTLA-4 molecule to the cell surface. Furthermore, the difference in expression of CTLA-4 between the studied groups of CLL patients upon ex vivo stimulation seems to confirm the observations of other authors that the response of leukaemic cells to CpG ODN stimulation is heterogeneous [35–37]. The reason for the different patterns of CTLA-4 molecule expression in response to ex vivo DSP30+rIL-2 stimulation in the studied groups of CLL patients requires further clarification. On the other hand, as the high level of CTLA-4 expression in CLL cells is one of the good prognostic factors [19], application of CpG ODN that reduces the surface expression of CTLA-4 in leukaemic cells as a therapeutic agent seems to be an unfavourable strategy for CLL patients.

Since CTLA-4 is involved in regulation of proliferation [12, 13, 28] and cell survival [29], an intriguing question is whether the peripheral blood leukaemia cells from CLL patients differ in terms of the proliferation activity and apoptosis before as well as after ex vivo stimulation regarding CTLA-4 expression. Analysis of the expression of Ki67 protein in freshly drawn lymphocytes as well as in cells cultured with DSP30 and rIL-2 showed no significant differences in proliferation activity of leukaemic cells from the two studied groups of CLL patients at each time point tested. With regard to freshly isolated CLL cells, we suggest that this lack of a marked difference in the median proportions of Ki67-positive cells between studied groups of CLL patients may result from the relatively low proliferation activity of the peripheral blood leukaemic lymphocytes in vivo. In fact, the vast majority of circulating CLL cells are arrested in the G0/G1 phase of the cell cycle [20, 38] and do not express Ki67 protein [39]. As regard proliferation activity after ex vivo stimulation, we assume that the observed lack of significant differences between studied groups of CLL patients may be caused by a gradual decline of CTLA-4 expression in the high CTLA-4 expressors following ex vivo stimulation to the level found in the low CTLA-4 expressors.

An interesting finding of the present study was that the studied groups of CLL patients also differ in terms of apoptosis following cell culture. We found that after 72 h of culture in medium alone, a lower proportion of CLL cells from the high CTLA-4 expressors became apoptotic compared to those from the low CTLA-4 expressors, which corresponded with the significant down-regulation of surface CTLA-4 expression only in the high CTLA-4 expressors. Accordingly, Mittal et al. [21] connected the down-regulation of CTLA-4 expression in CLL cells with the declined apoptosis of these cells. The authors reported that the decline of CTLA-4 expression in CLL cells led to decreased frequency of apoptotic cells as a result of increased expression of the anti-apoptotic molecule B cell lymphoma 2 (Bcl-2) observed at both the mRNA and protein levels.

One of the main aims of the present study was to investigate the effect of CTLA-4 blockade on the proliferation activity and apoptosis of CLL cells. As clinical trials with systemic administration of ipilimumab (fully human mAb directed against CTLA-4, formerly MDX-010) in CLL were recently performed [23], the results of these experiments not only broaden our knowledge about the role of the CTLA-4 molecule in the regulation of proliferation and survival of CLL cells, but also provide very important information from the clinical point of view. In the current study, we observed a significant increase in the median proportion of Ki67-positive leukaemic cells following CTLA-4 blockade, but only in the high CTLA-4 expressors. It is noteworthy that in the high CTLA-4 expressors, proliferation activity of leukaemic cells might be a result of two signals: one originating from the down-regulation of CTLA-4 expression following CpG ODN stimulation observed in this group only and the second one coming from blockage of the CTLA-4-mediated inhibitory signal. The relationship of CTLA-4 down-regulation in CLL cells with the increased proliferation of these cells measured by MTT and ^3^H-thymidine uptake assays as well as by Ki67 protein expression was recently observed [21]. Furthermore, in CLL cells with rapidly down-regulated expression of CTLA-4, a significant increase in the expression level of the transcription factor signal transducer and activator of transcription 1 (STAT1) as well as in its phosphorylation level was observed [21]. STAT1 is phosphorylated by the phosphorylated form of p38 mitogen-activated protein kinase (p38MAPK), which is a downstream effector of BCR crosslinking as well as TLR9 engagement by its ligands, CpG-ODNs [40, 41]. Recently, Comin-Anduix et al. [42] reported that PBMCs from patients with metastatic melanoma treated with tremelimumab (fully human IgG2 mAb against CTLA-4 in clinical development for patients with cancer) were characterised by an increased level of phosphorylated form of p38MAPK (pp38MAPK). Thus, as down-regulation of CTLA-4 expression results in the increase and phosphorylation of STAT1 in CLL cells [21] and CTLA-4 blockade may lead to an increased level of pp38MAPK [42], we cannot exclude that the increased proliferation activity in the high CTLA-4 expressors following CTLA-4 blockade might result from activation of STAT pathways. On the other hand, it still remains unsolved why, in the low CTLA-4 expressors, CTLA-4 blockade did not affect the proliferation activity of leukaemic cells; probably, the optimal level of CTLA-4 expression is needed.

Similarly, we observed the impact of CTLA-4 blockade on apoptosis of CLL cells. However, we found an opposite influence of CTLA-4 blockade on the frequency of apoptotic cells in studied groups of CLL patients regarding CTLA-4 expression. Among the patients, a high tendency to decrease in apoptotic cell frequency following CTLA-4 blockade was noted in the high CTLA-4 expressors. Consequently, we observed a markedly lower apoptosis rate in these patients compared to controls. In the low CTLA-4 expressors as well as in healthy individuals, a tendency to an increase in the median percentages of apoptotic cells was observed. Based on the mentioned study [21], we strongly suggest that quick down-regulation of CTLA-4 expression after blockade may result in increased expression of anti-apoptotic Bcl-2. On the other hand, blockade of CTLA-4 may result in inactivation of the phosphatidylinositol 3-kinase/protein kinase B (PI3K/Akt) pathway. It has been shown that CTLA-4 induces activation of the PI3K/Akt pathway, resulting in inactivation of the pro-apoptotic factor Bcl-2 antagonist of cell death (BAD) and upregulation of the survival factor B cell lymphoma-extra large (Bcl-X_L_) [29]. Thus, inactivation of the PI3K/Akt pathway following CTLA-4 blockade may block the inactivation of BAD and promote, in consequence, the apoptosis of cells. In the light of our findings, we can suppose that in the high CTLA-4 expressors, the pro-survival signal originating from the rapid down-regulation of CTLA-4 expression seems to prevail. Conversely, in the low CTLA-4 expressors and healthy individuals, where down-regulation of CTLA-4 expression was not observed, predominance of the pro-apoptotic signal resulting from inactivation of the PI3K/Akt pathway upon CTLA-4 blockade should not be excluded. Additional studies are needed to explain the mechanisms leading to a decrease or increase in the frequency of apoptosis of leukaemic cells following CTLA-4 blockade.

In summary, we have demonstrated for the first time the different pattern of ex vivo-stimulated CTLA-4 expression in low and high CTLA-4-expressing CLL groups. Furthermore, the rate of CTLA-4 expression seems to determine proliferation activity and apoptosis of CLL cells; CTLA-4 blockade in high CTLA-4 expressors induces pro-survival signals in CLL cells, indicating that the systemic administration of a CTLA-4 blocking antibody as immunotherapy might be an unfavourable strategy for these patients. In contrast, this form of immunotherapy might benefit CLL patients with low CTLA-4 expression on leukaemic cells (which predicts a poor clinical outcome), since CTLA-4 blockade does not affect proliferation and apoptosis of malignant B cells in this group. Therefore, CTLA-4 expression on B cells might distinguish CLL patients in whom systemic administration of a CTLA-4 blocking antibody for T cell effector function improvement is beneficial and safe.
